# Non-Radiation Based Early Pain Relief Treatment Options for Patients With Non-Small Cell Lung Cancer and Cancer Induced Bone Pain: A Systematic Review

**DOI:** 10.3389/fonc.2020.509297

**Published:** 2020-10-22

**Authors:** Anita J. W. M. Brouns, Ben H. De Bie, Marieke H. J. van den Beuken-van Everdingen, Anne-Marie C. Dingemans, Lizza E. L. Hendriks

**Affiliations:** ^1^ Department of Pulmonary Diseases, Zuyderland Medical Center, Sittard-Geleen, Netherlands; ^2^ Department of Pulmonary Diseases, GROW—School for Oncology and Developmental Biology, Maastricht University Medical Center+ (MUMC+), Maastricht, Netherlands; ^3^ Department of Anesthesiology, Maastricht University Medical Center+ (MUMC+), Maastricht, Netherlands; ^4^ Centre of Expertise for Palliative Care, Maastricht University Medical Center+ (MUMC+), Maastricht, Netherlands; ^5^ Department of Pulmonary Diseases, Erasmus MC, Rotterdam, Netherlands

**Keywords:** non-small cell lung cancer, bone metastases, cancer induced bone pain, pain relief, bisphosphonates, systematic review

## Abstract

**Introduction:**

Cancer induced bone pain (CIBP) is frequent in patients with non-small cell lung cancer (NSCLC). Radiation therapy continues to be the gold standard for treatment of painful bone metastases, however only a limited number of metastases can be irradiated. We evaluated non-radiation based early CIBP relief options in NSCLC through a systematic review.

**Methods:**

Systematic review including all prospective articles published between 01-1994 and 06-2020 on Pubmed, Cochrane Library and ClinicalTrials.gov database. Inclusion: non-radiation based trials evaluating CIBP early pain relief options (initially defined as pain score evaluated within two weeks, because of no randomized trials, later inclusion broadened to pain score evaluated within six weeks) in ≥10 NSCLC patients. Radioisotope trials were excluded as these treatments have interactions with systemic anticancer therapy.

**Results:**

188 articles were found; 10 articles (6 randomized controlled (4 double blinded), 1 phase II single-arm, and 3 prospective trials) fulfilled the inclusion criteria. Six of these trials consisted of ≥2 treatment arms, whereas the others were single-arm studies. In total, 554 NSCLC patients were evaluated in these trials. The included trials were very heterogeneous regarding evaluated treatment options, methods of pain measuring, and endpoints. No high-level evidence for specific early pain relief treatment options was found.

**Discussion:**

Non-radiation based studies evaluating treatment options to rapidly reduce CIBP in NSCLC are scarce. This systematic review shows that there is no high-level evidence to recommend a specific treatment for early pain relief. Future research should focus on early pain relief treatment options for CIBP in NSCLC.

## Introduction

Bone metastases are diagnosed in 24%–70% of patients with non-small cell lung cancer (NSCLC) during the course of the disease (1–3). Up to 80% of these patients experience cancer induced bone pain (CIBP) (4). Unfortunately, scarce data is available describing the severity of bone pain in patients with lung cancer; only the incidence of bone pain or usage of analgesics is reported (1–3, 5). In about one fifth of the patients, Quality of Life (QoL) worsens after a diagnosis of bone metastases (6). Furthermore, bone metastases are associated with lower overall survival (OS) (6).

Tumor invasion into bone causes osteoclast and osteoblast recruitment and modulation of genes and proteins involved in the bone microenvironment. Numerous factors are involved in the process of bone pain such as nociceptor activation and sensitization, ectopic sprouting of nerve fibers and central sensitization in the spinal cord and brain. Without treatment of the underlying disease and/or local treatment, no bone healing occurs in bone metastases, leading to a vicious circle of CIBP, central sensitization resulting in more pain, and the development of chronic bone pain (7). Therefore, early pain reduction is important.

Due to the high incidence, chronic character, and negative impact on QoL and OS, CIBP is an important issue that needs to be addressed in metastatic NSCLC. According to the World Health Organization (WHO) pain ladder, (bone) pain should first be treated with paracetamol and/or non-steroidal anti-inflammatory drugs (NSAIDs), followed if necessary by adding mild, and later strong opioids (8). The extended use of NSAIDs and opioids is associated with unwanted side effects (e.g., renal, hepatic or gastro-intestinal) (7). Especially in the older population (i.e., most patients with lung cancer), opioids can lead to neurological complaints such as dizziness or cognitive clouding, which in turn increases the likelihood of falling with the risk of for example bone fractures (7). Furthermore, several patients are reluctant to take opioids because of fear to become addicted, or because of the side effects (9). Radiotherapy is another effective treatment strategy for bone pain with a complete pain resolution in approximately 50% of the patients (10). Drawbacks of radiotherapy as treatment option are the time delay, as it takes up to 6 weeks before a maximum treatment effect is obtained (although ≥50% of responders have benefit within 1–2 weeks) and a frequently occurring pain flare-up in the first week after radiotherapy (10, 11). In addition, radiotherapy is only feasible in patients with a limited number of painful bone metastases.

The European Society for Medical Oncology (ESMO) guideline on metastatic NSCLC (2018) recommends denosumab or zoledronic acid in patients with NSCLC and bone metastases considered at high risk for skeletal related events (SREs) and with a life expectancy of >3 months (level of evidence I, grade of recommendation B) (12). This recommendation is based on the observation that bone targeted agents reduce SREs. Of note, pain scores are not included in the definition of SRE, although necessity for radiation because of painful bone metastases is included. For denosumab it was found that in patients with bone metastases and no/mild baseline CIBP, time to pain interference with daily life was longer compared with zoledronic acid. The ESMO advice is based on randomized phase III trials that included solid tumors (approximately 50% NSCLC) and early pain relief was not a primary objective of these trials (12). Trials including patients with bone metastases from prostate- or breast- or lung cancer (N=607 of which 1 NSCLC), which evaluated the effect of ibandronate (intravenous or oral) on bone pain showed pain relief within seven days after start of ibandronate (13–15). However, most of the patients received concomitant antineoplastic treatment, therefore a pain relief effect of the systemic anti-cancer therapy cannot be excluded and is it difficult to evaluate the therapeutic effect on CIBP of bisphosphonate therapy alone.

The ESMO guideline on bone health in patients with cancer (2020) states that multidisciplinary management (e.g., systemic treatments, radiation therapy, surgery and supportive care) is needed for effective treatment of metastatic bone disease. They suggest radiotherapy as treatment of choice in localized CIBP, but no specific treatment recommendations are made for diffuse CIBP (10). The National Comprehensive Cancer Network (NCCN) guideline on NSCLC and National Institute for Health and Care Excellence (NICE) flowcharts on lung cancer mention radiotherapy as pain relief option in CIBP (16, 17).

Survival is improving in patients with NSCLC, partly because of the survival benefit seen with immune checkpoint inhibitors for a large proportion of patients and partly due to the availability of tyrosine kinase inhibitors for the group of patients with an oncogenic driver. As it is possible that these patients live longer with CIBP, effective, preferably early pain reducing treatments might be more relevant. We performed a systematic review specifically focusing on early non-radiation based pain relief options for NSCLC patients with CIBP.

## Materials and Methods

### Search Strategy and Selection Criteria

A systematic search of the literature published between January 1994 and June 2020 was performed using the PubMed, the Cochrane Library and the ClinicalTrials.gov database. Published studies were identified using a search strategy based on the Patient-Intervention-Control-Outcome (PICO) method (shown in **Table 1** in the **Supplementary Material**) (18). PRISMA 2009 checklist for systematic reviews is shown in **Table 3** in the **Supplemental Material**. Our clinical question was to assess the efficacy of CIBP relief treatment options in patients with NSCLC. Initially, we defined early pain relief as pain reduction within two weeks. As we identified only one trial, and to be as inclusive as possible, we expanded the time to six weeks because in this period the maximum effect of radiotherapy occurs. We excluded radiotherapy because of the aforementioned drawbacks, and radioisotopes since the possible interaction with systemic treatment which is the mainstay of treatment for the majority of patients with NSCLC (19). The main inclusion criteria were 1) prospective trials focusing on treatment options for early pain relief, 2) inclusion of a minimum of 10 patients with NSCLC and with at least one bone metastasis. All inclusion criteria for this systematic review are summarized in **Table 2** in the **Supplementary Material**.

### Study Selection

Two authors (AB and BB) independently screened the titles of the selected studies and subsequently the abstracts of the eligible studies. The same authors independently examined the full texts of the selected articles regarding the inclusion criteria. Studies were included if they met the eligibility criteria. To complete the search, the references of all eligible articles were manually searched for additional relevant articles. Also, the excluded review articles were screened for relevant studies which were not represented in the original search. The entire search and selection were independently checked by a third reviewer (LH). In case of disagreement during study inclusion, consensus was sought.

### Data Selection

When available and applicable, the following data were extracted from eligible studies by one author (AB) and independently by another author (BB): year of publication, number of study arms, randomization method, duration of study and follow-up, histological diagnosis, intervention (i.e., type, dose, duration, route and frequency), method of pain score (e.g., bone pain inventory [BPI]), timing of pain score, efficacy of intervention on pain relief, whether results were specifically for NSCLC or for all included patients, and primary and secondary objectives of the trials. Final approval of the extracted data was performed by LH.

The Jadad scale was used to assess the methodological quality of the included trials (20). We did not perform a formal test of heterogeneity because of the heterogeneous type of trials included in the systemic review, with one third of the included trials being single arm (i.e., per definition high risk of bias).

## Results

### Study Selection

**Figure 1:**
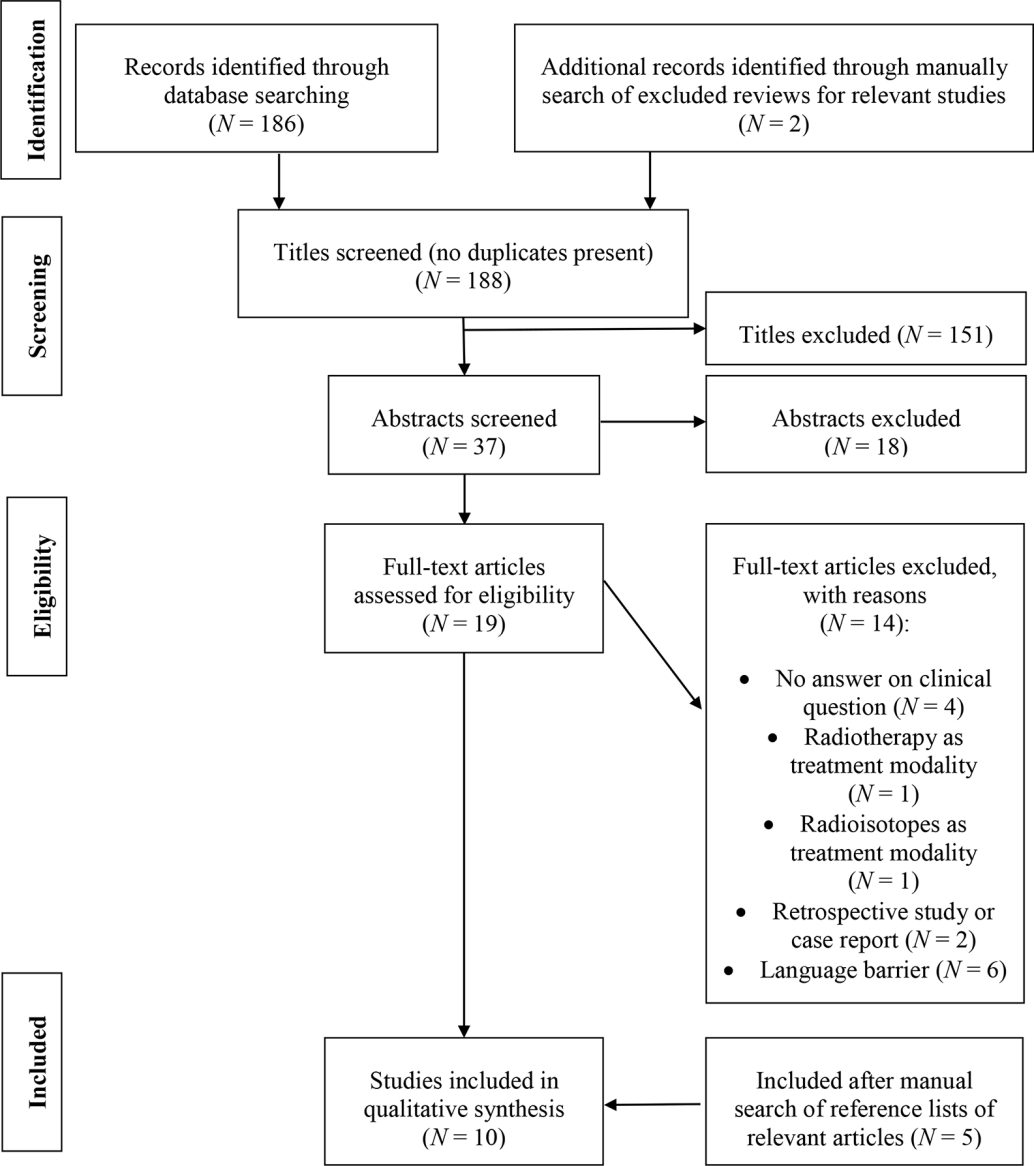
Flowchart for article selection.

The literature search identified 186 articles in total without duplicates. As mentioned in the inclusion criteria, reviews were excluded in the search strategy, but to broaden the search results these reviews were manually searched for relevant studies. After checking the reference list of the reviews identified with the systematic search, 2 additional relevant articles were included. Of these 188 articles, 151 were excluded because of non-relevant titles. 18 of the 37 remaining articles were excluded because they did not fulfill the inclusion criteria based on the abstract. After screening of the full text of the remaining 19 articles, 14 articles were excluded because of: no answer on the clinical question (N = 4), radiotherapy as treatment modality (N = 1), radioisotopes as treatment modality (N = 1), retrospective study or case report (N = 2), and a language barrier (e.g., Chinese, Japanese or Serbian language, N = 6). After manual search of the reference list of included articles 5 other relevant articles were included. The flowchart for article selection is shown in **Figure 1**.

### Description of Studies

One phase II trial (21), six randomized controlled trials (22–27) and three other prospective series (28–30) were included in this review. The randomized controlled trials were double-blinded in four trials (24–27) (of which one was placebo-controlled) and open label in two (22, 23). Three studies were single-arm (21, 28, 29) six were 2-arm (22, 24–27, 30) and one 3-arm (23). In the study of Zarogoulidis et al., the selection was made on the presence of bone pain (30). The main characteristics of the included studies are shown in **Table 1**. Four studies included only patients with NSCLC, with a minimum of one bone metastasis (21, 22, 28, 30). The other studies included also patients with CIBP caused by bone metastases of solid malignancies (256 out of 978 patients in total had NSCLC) (23–27, 29). The number of patients with NSCLC enrolled in the studies ranged from 14 (24, 28) to 144 (30), leading to in total 554 patients with NSCLC included in this review. Only in five trials patients received pain modifying therapy alone (23–25, 27, 28), in all other trials, other anticancer treatment (mainly chemotherapy) was given (21, 22, 26, 29, 30). The inclusion criteria were quite similar across the included trials. The only difference was the treatment history of the patients, e.g., pre-treated with radiotherapy (28), chemonaïve (29) and presumably chemonaïve (22, 30) and pre-treated with chemotherapy and/or radiotherapy (21, 26). In the other trials, no information about previous therapy is provided (23–27). The exclusion criteria concerning comorbidities were comparable among eight studies (21–27, 29). In two other studies, no exclusion criteria were mentioned (28, 30).

**Table 1:** Main characteristics of included studies.

Study (year)	Trial type	Jadad score	Total pts/NSCLC pts	Treatment arm	Comparator arm	Median follow-up (months)	Primary objective(s)	Secondary objective(s)
Yousef and Alzeftawy (27)	Phase NR, prospective Randomized, 2 arms, single center	4	19/100	Sublingual fentanyl^%^	Piroxicam fast-dissolving tablets^%^	1	Pain intensity reduction, frequency of BTP attacks, onset of pain relief	Functional interference items of BPI
Liu et al. (23)	Phase NR, prospective Randomized 3 arms, single center	3	95/342	Diclofenac + celecoxib + morphine sulfate*	Diclofenac + morphine sulfate* Celecoxib + morphine sulfate*	NR	VAS score, remission rate, breakthrough pain, dose of morphine sulfate	Tox
Davidov (21)	Phase NR, prospective Single-arm, single center	0	53/53	Cycles of Gem (1,250 mg/m2) on d1+8, CDDP (80 mg/m2) on d1 and ZOL 4mg IV once Q3-4w	None	NR 14 (mean)	Efficacy, safety of ZOL IV	NA
Sjöland et al. (26)	Phase NR, prospective Randomized, double, 2 arms, multicenter	3	39/152	Flexible-dose pregabalin^$^ as add-on to stable opioid analgesic therapy	Placebo as add-on to stable opioid analgesic therapy	NR	DAAC from baseline in daily worst pain on first day stable dose to day 28	DAAC for average pain, NRS sleep interference scores, change in mBPI-sf, HADS change, change in PGIC score
Sima et al. (25)	Phase NR, prospective Randomized 2 arms, multicenter	4	75/246	1–3 Oxycodone/paracetamol (5/325 mg) Q6H d1-3^#^ + morphine/ fentanyl patches	Placebo + morphine/ fentanyl patches	NR	PID	Breakthrough pain, rescue morphine consumption
Yoh et al. (29)	Phase II Single-arm, single center	0	35/35	1–4 cycles of CDDP (80 mg/m2), D (60 mg/m2) and ZOL 4mg IV on d1 Q3-4w	None	16	Feasibility of CDDP, D and ZOL	Tox, SRE, pain scores, best ORR, OS
Francini et al. (22)	Phase NR Randomized 2 arms, single center	2	55/55	ZOL 4 mg IV Q4w and CTX (CDDP/GEM, CDDP/VBN, CDDP/GEM + BEV)	IBA 50 mg q.d. and CTX (CDDP/GEM, CDDP/VBN, CDDP/GEM + BEV)	NR	Effect ZOL and IBA on s-CTX and B-ALP	Bone pain, SRE, time to first SRE, TTP of BM, OS
Zarogoulidis et al. (30)	Phase NR, prospective Non-randomized, 2 arms, single center	0	144/144	Pts with bone pain: ZOL 4mg IV Q4w and 1–8 cycles of D (100 mg/m2), carbo (AUC = 6) In case of response to CTX: 50Gy RTX to primary site between 2-3rd cycle	Pts without bone pain: 1–8 cycles of D (100 mg/m2), carbo (AUC = 6) In case of response to CTX: 50Gy RT to primary site between 2-3rd cycle	NR	TTP, OS	Bone lesion response, change in BPI, change in biochemical markers of bone resorption
Rodríguez et al. (24)	Phase NR, prospective Randomized, 2 arms, multicenter	4	14/113	Dexketoprofen trometamol 25 mg q.i.d.	Ketorolac 10 mg q.i.d.	NR	Pain intensity at d 7 +1	Pain intensity at d 3 ± 1, %pts reaching PID ≥20mm from baseline/pain level <30 mm on VAS at d7, QoL, analgesic efficacy, %pts withdrawn from study, %pts needed rescue medication
Gangi et al. (28)	Phase NR Observational, prospective study, single center	0	14/25	Percutaneous injection of 3–25 ml ethanol 95%	None	NR	Pain score	NA

NR, not reported; BTP, break through pain; VAS, Visual Analogue scale; Tox, toxicity; Gem, gemcitabine; CDDP, cisplatin; D, docetaxel; ZOL, zoledronic acid; IV, intravenous; d, day; Q, every; SRE, Skeletal Related Event; ORR, objective response rate; OS, overall survival; NA, not applicable; DAAC, duration-adjusted average change; NRS, numeric rating scale; mBPI-sf, modified Brief Pain Inventory Short Form; HADS, Hospital Anxiety and Depression Scale; PGIC, Patient Global Impression of Change; Q6H, every six hours; PID, pain intensity difference; w, week; CTX, chemotherapy; VBN, vinorelbine; BEV, bevacizumab; IBA, ibandronate; q.d., once a day; s-CTX, serum C-telopeptide of collagen type I; B-ALP, bone-alkaline phosphatase; TTP, time to progression; Pts, patients; BM, bone metastasis; carbo, carboplatin; AUC, area under the curve; RT, radiotherapy; BPI, bone pain inventory.

*Dosage of painkillers was as follows: diclofenac 100 mg/12h; celecoxib 400mg/day; morphine sulphate 10mg/12h, with a reduction of 50% or addition of 25% each time until the VAS score was <5.

^$^Dosage of pregabalin was as follows: 100, 150, 300, or 600mg/day.

^#^The amount of placebo or oxycodone/paracetamol tablets was titrated step by step based on the pain assessment, up to 12 tablets per day maximum.

^%^Doses were adjusted individually. The effective dose was defined as the dose needed to control BTP (pain reduction by 50% in each pain episode without the occurrence of relevant adverse events).

The primary objectives of the included trials varied from efficacy and safety of combined treatment of chemotherapy and zoledronic acid (21, 29) effects of zoledronic acid on bone resorption or formation markers (22), efficacy of treatment on time to progression (TTP), OS (30), reduction in pain intensity (24–27), frequency of breakthrough pain (24, 27), remission rate (24), dose of morphine sulfate (24), and duration-adjusted average change (DAAC) from baseline in the daily NRS worst pain score (26). Six studies had (reduction in) pain score as primary or secondary objective (23–28). Three studies assessed bone pain or change in BPI from baseline as secondary objectives (22, 29, 30). The study of Davidov et al. measured pain scores as an exploratory objective (21). **Table 1** provides a detailed summary of all outcome variables of the included studies.

### Results of Individual Studies

#### Method and Timing of Pain Score

The studies of Yoh et al. and Zarogoulidis et al. used the BPI as method of measuring pain score (29, 30). In Zarogoulidis’s study, the BPI was scored each clinical visit (the interval between study visits was not further specified) (30), whereas in Yoh’s et al. study this assessment was performed at baseline and after six weeks of treatment (29). Three studies used the visual analogue scale (VAS) expressed in figures or millimeters as method of measuring pain score (23, 24, 27). The pain assessments took place within the first week of treatment (23, 24, 26, 27) till four weeks (23, 26, 27). Two other studies were not focused on a direct pain score, they evaluated pain response indirectly with the use of analgesics (21, 28). This was evaluated after one and three cycles of chemotherapy (e.g., first measurement at three-four weeks after start of anticancer treatment) (21) and within 48 h after the intervention with a re-evaluation after two weeks (28). One study used the McGill-Melzack pain score, which was performed at baseline and after one and three months of treatment (22). The numeric rating scale (NRS) as method of measuring pain score was used by two trials (25, 26), one double blind randomized trial only recorded till day three of treatment (25), whereas the other recorded daily till day 35. Details on methods and timing of pain score are in **Table 2**.

**Table 2:** Overview of reported items on early bone pain relief.

Study (y)	Method of pain score	Timing of pain score	Pain score on baseline	Efficacy treatment on pain	Results specified for NSCLC only	Duration of response
Yousef and Alzeftawy (27)	VAS	3d, 1, 2, 3, 4 w	Fentanyl: 8.09 ± 0.75 Piroxicam: 8.3 ± 0.75	At 1 m Fentanyl: 3.37 ± 0.74 Piroxicam: 3.47 ± 0.76	No	NR
Liu et al. (23)	VAS	1^st^, 2^nd^, 4^th^ w after treatment	Dicl+ Cele: 8.48 ± 1.06 Dicl: 8.53 ± 1.06 Cele: 8.50 ± 1.06	At d 28^2^ Dicl+ Cele: 2.40 ± 1.20 Dicl: 3.50 ± 0.70 Cele: 3.40 ± 0.70	No	NR
Davidov (21)	NR	After 1 and 3 cycles	NR	*After 1 cycle:* 5/53 pts reduced their analgesic need, 14/53 pts needed more pain medication, 34/53 pts showed no change in pain medication	Yes	NR
Sjöland et al. (26)	NRS	Each day	NR	Mean change DAAC pregabalin: -1.53 (1.81) Mean change DAAC (SD) placebo: -1.23 (1.74)	No	NR
Sima et al. (25)	NRS	d 1–3	Placebo: 5.3 Oxycodone/ paracetamol: 5.2	At d 3 PID placebo: 0.3 Oxycodone/ paracetamol: 1.5	No	NR
Yoh et al. (29)	BPI	Baseline, after 6 w	mean BPI 2.6 ± 0.2	mean BPI 1.0 ± 0.3 at 6 w (*p*<0.0001)	Yes	NR
Francini et al. (22)	McGill-Melzack pain questionnaire	Baseline, after 1 and 3 m	ZOL 1.98 ± 1.12, IBA 1.88 ± 0.89	“Trend for more rapid decrease in bone pain score in favor of ZOL”	Yes	NR
Zarogoulidis et al. (30)	BPI	Each clinical visit	78 pts ≤ 4, 8 pts 4 - 6, 1 pt > 8	“no significant difference between treatment arms in pain effect of ZOL compared to baseline”	Yes	NR
Rodríguez et al. (24)	VAS in millimeters	d 3 ± 1, d 7 + 1	Dexketoprofen: 69 ± 15 Ketorolac: 75 ± 16	At d 7 Dexketoprofen: 32 ± 24 Ketorolac: 40 ± 30	No	NR
Gangi et al. (28)	Indirect measured by scale according reduction of opiate analgesics^1^	<48h after intervention, re-evaluation after 2w	“Pain relief insufficient after treatment with opiate analgesics and RTX and/or CTX”	55% of pts score ≥3, 74% of pts score ≥2, 26% of pts score1^1^	No	10-27 w

VAS, Visual Analogue scale; w, weeks; Dicl, diclofenac; Cele, celecoxib; d, days; NR, not reported; pts, patients; NRS, numeric rating scale; BPI, brief pain inventory; m, months; RTX, radiotherapy; CTX, chemotherapy.

^1^Scale consists of the following items: Score 4: complete relief (opiate analgesic drugs no longer necessary), score 3: very good but incomplete relief (75% reduction of analgesic requirement), score 2: good relief (25-50% reduction of analgesic requirement), score 1: little of no relief (<25% reduction or no change of analgesic requirement).

^2^Gr 1: diclofenac and celecoxib, Gr 2: diclofenac, Gr 3: celecoxib.

^3^VAS scores at day 7 and 14 are shown in the original article, they showed superiority in pain reduction the diclofenac and celecoxib group.

#### Efficacy of Treatment on Pain and Duration of Response

Five studies (including 217 patients with NSCLC, out of 554 patients included in total) showed a significant treatment effect on pain score (23–25, 27, 29). One double blind randomized controlled trial, including 14 patients with NSCLC out of 113 included patients (10%) evaluating dexketoprofen trometamol versus ketorolac showed superior of the former on pain rating index (secondary outcome, p = 0.04) (24). One open label, randomized controlled trial, showed that diclofenac combined with celecoxib and morphine sulfate was superiority to NSAID monotherapy combined with morphine sulfate in CIBP reduction, measured with VAS (average VAS score at 28 days: 2.40 ± 1.20 vs 3.50 ± 0.70 (diclofenac monotherapy plus morphine) or 3.40 ± 0.70 (celecoxib monotherapy plus morphine), p = 0.006) (23). Another double blind randomized controlled trial, including 75 patients with NSCLC out of 246 included patients (30%) showed an additional effect of the combination of short acting oxycodone/paracetamol versus placebo, added to standard long-acting opioids on reducing bone pain (pain intensity difference (PID) after three days in the placebo group 0.3, compared with 1.5 in the oxycodone/paracetamol group, p<0.001) (25). One, double blind randomized controlled, trial, including 19 patients with NSCLC out of 100 included patients (19%), evaluating fentanyl versus piroxicam for CIBP reduction, reported for both drugs a significant decrease in VAS score at 1 month. No significant difference in efficacy was found between the treatment arms (27). The only study with bisphosphonates, which found a significant effect of treatment on pain score was the single-arm study of Yoh et al. (29). They showed that treatment of both chemotherapy and zoledronic acid reduced pain score at six weeks compared to baseline. In another study, no significant difference in pain effect of zoledronic acid between the treatment arms (docetaxel and carboplatin +/- zoledronic acid) was observed (30). Another double blind randomized controlled trial, including 39 patients with NSCLC out of 152 included patients (26%), reported a non-significant effect of pregabalin treatment on pain compared placebo (DAAC from baseline in the daily NRS worst pain score -1.53 vs. -1.23). The study of Francini et al. showed a trend for more rapid decrease in bone pain score at one month in favor of zoledronic acid compared to oral ibandronate (22). Davidov et al. found only a reduced analgesic need in five out of 53 patients (10%), whereas most of the patients (34 out of 53 patients [64%]) had no change in pain medication after one treatment cycle (21). One study showed a reduction of minimal 25%–50% of analgesic requirement in 74% of the patients and 55% of the patients had a reduction of 75% of analgesic requirement after treatment with ethanol injections (28). The duration of treatment response was only reported in one study and was ten to 27 weeks (28).

Of note, none of the studies including patients with different primary tumor histologies reported results for the subgroup of patients with NSCLC. **Table 2** provides an overview for reported items on bone pain relief.

### Discussion

CIBP is a clinically relevant problem in metastatic NSCLC due to the high prevalence of bone metastases, the chronic character and the negative impact on QoL and OS (6). Survival is improving for NSCLC: five-year survival improved from 5% to 16%–31% for patients without targetable mutations, treated with immune checkpoint inhibitors and five-year survival rates are over 40% in patients with an *EGFR* mutation or *ALK* fusion (31–37). It is possible that some of these patients survive a prolonged time with CIBP that impairs QoL, making effective pain reducing strategies necessary.

To obtain more insight in possible treatment options for early pain relief in patients with NSCLC with bone metastases and CIBP, we performed a systematic review on this topic excluding radioisotopes and radiotherapy for the reasons mentioned above. The initial scope of this review was early pain relief (pain relief evaluated within two weeks of start of treatment), but this resulted in limited number of eligible trials. To be more inclusive, we broadened the time of “early pain relief” to a maximum of six weeks. Even then, only ten studies were eligible. Of note, the included trials were very heterogeneous regarding treatments evaluated, primary endpoints, methods of pain measurement and timing of assessment. Importantly, the randomized trials included patients with different histologies, and patients with NSCLC only comprised a subgroup in these randomized trials (554 [44%] of included patients). Importantly, not all treatments evaluated are comparable with recommended pain treatment in clinical guidelines. For example, according to international and national guidelines for breakthrough cancer pain, shorting-acting morphine should be added to standard dose long-acting morphine to treat breakthrough pain (38). Three of the included studies indeed underscore the importance of adding breakthrough medication to continuous release medication (23, 25, 27). As the comparator arms of these trials included a non-optimal treatment according to current guidelines, the results found in these trials have limited value in daily clinical practice. Another study excluded patients previously or currently treated with a scheduled regimen of painkillers, except acetaminophen and acetylsalicylic acid, which is also not according to the WHO pain ladder (8, 24).

In most other studies (21, 22, 29, 30), systemic therapy and pain relief therapy were administered concurrently, therefore conclusions on the specific efficacy of pain relief therapy were difficult as it cannot be excluded that the systemic therapy also causes a reduction in pain. For zoledronic acid, only one study showed an early pain reduction, but this pain reduction disappeared at three months despite continuous bone targeted agent use (22). While out of the scope of this review, information on long-term pain reduction is also particularly important. Only two studies provided follow-up of more than one year (21, 29). As CIBP is a chronic problem, it is also of interest to know information about the pain efficacy in the long term. However, only the studies of Davidov and Yoh had a follow-up of more than a year (21, 29). Duration of pain response and the recurrence rate of CIBP was lacking.

What are other possible treatment options for CIBP in NSCLC? The first step to achieve early pain relief in CIBP is analgesics according the WHO pain ladder (8). This advice is based on general pain management recommendations for patients with cancer. As was found in this review, opioids are indeed effective in the treatment of CIBP. Palliative radiotherapy is frequently used in the treatment of CIBP, because of the high response rate (around 85%). Drawbacks are the possibility of a pain flare-up and the limited use in multiple painful bone metastases (10). Besides that there are disparities in the access to radiotherapy facilities in high and low-income countries. For example, in Central Africa 0.05 machines are available per million people versus 11.4 machines in North-America (39). Furthermore, even if there is access, older, multi-fractionated radiotherapy schedules for treatment of painful bone metastases are often used, instead of the recommended single-fraction radiotherapy, as was shown in a survey on radiation facilities in African countries (40). This further limits the access to (up-to-date) radiotherapy facilities and strengthens the need for other early pain relief options for patients with NSCLC”. Bisphosphonates and denosumab are also used to tread CIBP. Trials including patients with breast and prostate cancer with uncontrolled CIBP indeed showed a reduction in pain scores with (loading doses) of bisphosphonates. However, data in NSCLC is scarce and results found in our systematic review do not show a clear reduction in CIBP in NSCLC. After our search, the NVALT-9 trial was published, and in contrast to the studies including patients with breast- or prostate cancer, loading doses of ibandronate did not lead to rapid bone pain relief in patients with NSCLC and uncontrolled bone pain (41). Denosumab was compared with zoledronic acid in a randomized phase III trial (1596 patients with solid tumors and at least one bone metastasis, 702 patients had NSCLC, patients with breast or prostate cancer were excluded). Primary endpoint was time to first on-study SRE, pain worsening was one of the other endpoints. Denosumab significantly delayed the time to pain worsening (HR, 0.83; 95% confidence interval, 0.71–0.97) in patients with no/mild baseline pain, compared to zoledronic acid (42). Results regarding early pain reduction in patients with baseline CIBP are not available. Unfortunately, the recently published randomized phase III Splendour trial, including only patients with advanced NSCLC (inclusion irrespective of presence of bone metastases), did not report data on the effect of denosumab on pain relief in the subgroup of patients with painful bone metastases (43). The trial design was to evaluate whether the addition of denosumab to standard first-line treatment improved OS; the primary endpoint was not met (12).

To the best of our knowledge, there is no explanation why for example bisphosphonates showed a reduction of CIBP in some malignancies but not in lung cancer (13–15, 41, 44, 45). Possible explanations are; differences in tumor histology/biology or bone metastasis metabolism [although bone turnover markers are comparable between for example breast and lung cancer (46)], which leads to different response on bone pain relief options, are probably the most obvious. Also the usage of different concomitant (systemic) therapies, which differs among malignancies, could strengthen pain control (47). Therefore, specific recommendations for (bone) pain relief are needed for different malignancies and findings cannot be extrapolated.

Radioisotopes (e.g., samarium, strontium, and rhenium) are an alternative treatment for CIBP. Radioisotopes have a rapid onset of action, but data on NSCLC are limited and consist only of subgroup analyses (19). Zoledronic acid combined with radioisotopes is another treatment option. The efficacy of adding a radioisotope (choice at discretion of investigator) to zoledronic acid was evaluated in the randomized phase III RTOG 0517 trial (26/262 included patients had lung cancer). (48). Primary endpoint was time to SRE development, pain control was a secondary endpoint. Only patients with stable or no bone pain were included. As a subgroup of patients did not have CIBP, and one of the treatment arms consisted of radioisotopes, we excluded this study in our article selection. The addition of radioisotopes resulted in superior pain control at one month, compared with zoledronic acid alone (median pain score of 0 versus 1, p=0.02). Subgroup analysis regarding the primary tumor histology or the presence of baseline CIBP were not performed (48). Because the relatively short duration of action of radioisotopes, it is expected that this treatment must be repeated several times if the patient has a prolonged survival.

Immune checkpoint inhibitors have become standard of care treatment for most patients with advanced NSCLC and result in durable responses in a subgroup of patients. For the subgroup of patients with oncogenic drivers, tyrosine kinase inhibitors often result in early and prolonged responses. For both classes of drugs, effects on CIBP have not been specifically reported. It is possible that in some patients, immune checkpoint inhibitors will not be very active in pain relief for CIBP, as Schmid et al. reported that efficacy of immunotherapy depends on the metastatic location: the treatment efficacy is less in bone lesions compared to lymph nodes (49). However, CIBP related outcomes have not been reported. Denosumab in combination with nivolumab, a programmed death-1 (PD-1) inhibitor, is currently under evaluation in patients with NSCLC and bone metastases (NCT03669523) with the overall response rate as primary outcome measurement. Time to first SRE is one of the secondary outcome measurements but there is no specific focus on pain relief. A phase II study with AL2846, a multi-target tyrosine kinase receptor inhibitor versus zoledronic acid in bone metastasized NSCLC (NCT04325776) is not yet recruiting. The primary endpoint is time to first SRE, and effectiveness of improving average daily pain (not specifically CIBP) is one of the secondary outcomes. Another, not yet recruiting, phase IV, study is zoledronic acid combined with radiotherapy for bone metastasis of NSCLC (NCT02480634). The primary outcome of this study is the percentage of patients who reach objective bone pain response.

Experimental studies in animal models of CIBP have shown alterations in, e.g., astrocytes or in the sphingolipid metabolism in the spinal cord or showed the importance of connexins in the cell-cell communication with probable effects on CIBP. Recently, different studies focused on therapeutic options to block or alter these pathophysiological changes. Blockade of interleukine-6 signaling is promising as it could lead to prevention or delay of bone remodeling as well as decreased pain intensity (4).

Some possible drawbacks for this systematic review exist. A point for discussion could be the chosen definition of early pain reduction (pain reduction within six weeks). We chose this upper limit to be as inclusive as possible to include treatment options that resulted in pain reduction within a relatively short term. Of note, for pain reduction treatment options within a shorter time frame (e.g., one or two weeks), even less data is available. As discussed above, bisphosphonates have different activity on early CIBP reduction in breast- and prostate cancer compared with NSCLC, we did not broaden our inclusion criteria to include other tumor types (13–15, 41).

Furthermore, as expected with over half of the included trials being single arm and/or not blinded and as shown by the Jadad score the methodological quality of most included trials is poor. Last, we did not include a formal test of heterogeneity, as only very heterogeneous trials met our inclusion criteria.

### Conclusion

In conclusion, despite the frequent occurrence of CIBP combined with the negative effects on QoL and OS, literature on the optimal treatment of CIBP in NSCLC is lacking. Most of the recommendations given in current guidelines are mainly based on data obtained in other tumors such as breast and prostate. Therefore, randomized trials evaluating treatment options with early pain relief for CIBP are necessary in lung cancer patients.

## Data Availability Statement

No datasets were generated or analyzed for this study.

## Author Contributions

Conceptualization: AB, BB, MB-E, LH. Formal Analysis: AB, BB. Writing—Original Draft Preparation: AB. Writing—Review and Editing: BB, MB-E, A-MD, LH. All authors agree to be accountable for the content of the work. All authors contributed to the article and approved the submitted version.

## Conflict of Interest

A-MD has taken part on an advisory board for Roche.

The remaining authors declare that the research was conducted in the absence of any commercial or financial relationships that could be construed as a potential conflict of interest.
