# Supplementary material for: Non-Radiation Based Early Pain Relief Treatment Options for Patients With Non-Small Cell Lung Cancer and Cancer Induced Bone Pain: A Systematic Review
Source: Front Oncol. 2020 Oct 22;10:509297. doi: 10.3389/fonc.2020.509297 (PMC7642688; doi:10.3389/fonc.2020.509297)
Supplement: Supplementary file 1 [file Table_1.docx]

Supplementary Material

# Table 1: Search Strategy based on PICO method.

| **PICO** |  | **Free search terms** |  | **MESH terms** |
| --- | --- | --- | --- | --- |
| **Patient** |  | Carcinoma Non-Small-Cell Lung  Carcinoma non small cell lung  Non-small-cell lung carcinoma  Non-small-cell lung cancer  NSCLC  Lung neoplasm  Lung neoplasms | OR | Carcinoma, Non-Small-Cell Lung  Lung neoplasms |
|  | AND | Distant metastasis  Distant metastases  Bone metastasis  Bone metastases  Stage IV  Metastatic disease | OR | Neoplasm metastasis |
|  | AND | Pain  Neuropathic pain  Palliative care  Cancer palliative therapy  Cancer pain  Bone pain | OR | Musculoskeletal pain  Nociceptive pain  Cancer pain  Neuralgia  Pain  Acute pain  Palliative care |
| **Intervention** | AND | Non opioids  Paracetamol  Acetaminophen  APAP  Non steroidal anti inflammatory drugs  NSAID  Morphine  Opioid  Antiepileptic  Anticonvulsant  Antidepressant  Anti-depressant  Tricyclic antidepressant  Selective serotonin and noradrenalin reuptake inhibitor  Selective serotonin reuptake inhibitor Serotonin inhibitor  N methyl d aspartate receptor antagonist  NMDA receptor antagonist  Corticosteroid  Glucocorticoid  Bisphophonate  Disphophonate  Denosumab  Cannabinoid  Cannabis sativa  Cannabis  Local anesthetics  Lidocaine  Capsaicin  Botulinum toxin A  Botulinum toxin  BTX-A  Aceclofenac  Benzydamine  Diclofenac  Ibuprofen  Indometcin  Meloxicam  Naproxen  Piroxicam  Tiaprofenic acid  Buprenorphine  Fentanyl  Hydromorphone  Oxycodone  Tapentadol  Tramadol  Gabapentin  Pregabalin  Valproate  Valproic acid  Phenytoin  Carbamazepine  Topiramate  Levetiracetam  Oxcarbazepine  TCA  SNRI  SSRI  Nortriptyline  Duloxetine  Imipramine  Venlafaxine  Bupropion  Citalopram  Clomipramine  Desipramine  Fluoxetine  Mirtazapine  Paroxetine  Sertraline  Ketamine  Memantine  Amantadine  Methadone  Cortisone  Dexamethasone  Fludrocortisone  Hydrocortisone  Prednisolone  Prednisone  Triamcinolone  Alendronic acid  Alendronate  Ibandronic acid  Ibandronate  Pamidronic acid  Pamidronate  Zoledronic acid  Clodronic acid  Zoledronate  Ropivacaine  Bupivacaine  Levobupivacaine  Risedronic acid  Lamotrigine  Amitriptyline  Doxepin  Dextromethorphan | OR | Analgesics, non-narcotic  Buprenorphine  Fentanyl  Hydromorphone  Oxycodone  Tapentadol (supplementary concept)  Tramadol  Methadone  Anticonvulsants  Gabapentin (supplementary concept)  Pregabalin  Valproic acid  Phentoin  Carbamazepine  Lamotrigine (supplementary concept)  Topiramate (supplementary concept)  Antidepressive agents,  Antidepressive agents, tricyclic  Antidepressive agents, second-generation  Adrenergic uptake inhibitors  Serotonin and noradrenaline reuptake inhibitors  Serotonin uptake inhibitors  Imipramine  Venlafaxine hydrochloride  Bupropion  Citalopram  Clomipramine  Desipramine  Fluoxetine  Mirtazpine (supplementary concept)  Paroxetine  Sertraline |
| **Comparator** |  | Not specified in search strategy in order to include single arm studies |  |  |
| **Outcome** | AND | Acute pain relief  Pain intensity  Pain control  Pain measurement  Pain response | OR | Pain measurement  Pain Management |

Abbreviations: NSCLC, non-small-cell lung cancer; APAP, acetaminophen; NSAID, non-steroidal anti-inflammatory drugs, NMDA, N-methyl-D-aspartate; BTX-A, botulinum toxin-A.

# Table 2: inclusion criteria.

| **Criterion** | **Definition** |
| --- | --- |
| Subjections included | Human only. |
| Language | No restrictions. |
| Article type | Original articles; reviews excluded. |
| Study phase | No restrictions, but retrospective series were excluded. |
| Year of publication | 1994 - September 2018. |
| Site of primary tumor | NSCLC with at least one bone metastasis, at least 10 NSCLC patients included in the study. |
| Age | ≥ 18 years. |
| Treatment | No restrictions on treatment for NSCLC. Treatment for pain should be focused on acute pain relief. All pain relief options, except radiotherapy and radioisotopes, are allowed. |
| Follow-up period | No lower or upper limit. |
| Outcome | Efficacy of analgesics on early pain relief (< 6 weeks) in patients with bone metastasized NSCLC. |

Abbreviations: NSCLC, non-small-cell lung cancer.

**Table 3 - PRISMA 2009 checklist for systematic reviews**

| **Section/topic** | **#** | | **Checklist item** | | **Reported on page #** |
| --- | --- | --- | --- | --- | --- |
| **TITLE** |  | |  | |  |
| Title | 1 | | Identify the report as a systematic review, meta-analysis, or both. | | 1. |
| **ABSTRACT** |  | |  | |  |
| Structured summary | 2 | | Provide a structured summary including, as applicable: background; objectives; data sources; study eligibility criteria, participants, and interventions; study appraisal and synthesis methods; results; limitations; conclusions and implications of key findings; systematic review registration number. | | 1-2. |
| **INTRODUCTION** |  | |  | |  |
| Rationale | 3 | | Describe the rationale for the review in the context of what is already known. | | 2-3. |
| Objectives | 4 | | Provide an explicit statement of questions being addressed with reference to participants, interventions, comparisons, outcomes, and study design (PICOS). | | 3, Table 1 Supplementary Material. |
| **METHODS** |  | |  | |  |
| Protocol and registration | 5 | | Indicate if a review protocol exists, if and where it can be accessed (e.g., Web address), and, if available, provide registration information including registration number. | | NA |
| Eligibility criteria | 6 | | Specify study characteristics (e.g., PICOS, length of follow-up) and report characteristics (e.g., years considered, language, publication status) used as criteria for eligibility, giving rationale. | | 3-6, Table 1. |
| Information sources | 7 | | Describe all information sources (e.g., databases with dates of coverage, contact with study authors to identify additional studies) in the search and date last searched. | | 3. |
| Search | 8 | | Present full electronic search strategy for at least one database, including any limits used, such that it could be repeated. | | Table 1 Supplementary Material. |
| Study selection | 9 | | State the process for selecting studies (i.e., screening, eligibility, included in systematic review, and, if applicable, included in the meta-analysis). | | 3-4. |
| Data collection process | 10 | | Describe method of data extraction from reports (e.g., piloted forms, independently, in duplicate) and any processes for obtaining and confirming data from investigators. | | 3-4. |
| Data items | 11 | | List and define all variables for which data were sought (e.g., PICOS, funding sources) and any assumptions and simplifications made. | | 4. |
| Risk of bias in individual studies | 12 | | Describe methods used for assessing risk of bias of individual studies (including specification of whether this was done at the study or outcome level), and how this information is to be used in any data synthesis. | | 4. |
| Summary measures | 13 | | State the principal summary measures (e.g., risk ratio, difference in means). | | NA. |
| Synthesis of results | 14 | | Describe the methods of handling data and combining results of studies, if done, including measures of consistency (e.g., I^2^_)_ for each meta-analysis. | | NA. |
| **Section/topic** | | **#** | | **Checklist item** | **Reported on page #** |
| Risk of bias across studies | | 15 | | Specify any assessment of risk of bias that may affect the cumulative evidence (e.g., publication bias, selective reporting within studies). | 3, 8. |
| Additional analyses | | 16 | | Describe methods of additional analyses (e.g., sensitivity or subgroup analyses, meta-regression), if done, indicating which were pre-specified. | NA. |
| **RESULTS** | |  | |  |  |
| Study selection | | 17 | | Give numbers of studies screened, assessed for eligibility, and included in the review, with reasons for exclusions at each stage, ideally with a flow diagram. | 4-5, Figure 1. |
| Study characteristics | | 18 | | For each study, present characteristics for which data were extracted (e.g., study size, PICOS, follow-up period) and provide the citations. | 4-5, Table 1 + 2. |
| Risk of bias within studies | | 19 | | Present data on risk of bias of each study and, if available, any outcome level assessment (see item 12). | NA. |
| Results of individual studies | | 20 | | For all outcomes considered (benefits or harms), present, for each study: (a) simple summary data for each intervention group (b) effect estimates and confidence intervals, ideally with a forest plot. | 5, Table 2. |
| Synthesis of results | | 21 | | Present results of each meta-analysis done, including confidence intervals and measures of consistency. | NA. |
| Risk of bias across studies | | 22 | | Present results of any assessment of risk of bias across studies (see Item 15). | 3, 8. |
| Additional analysis | | 23 | | Give results of additional analyses, if done (e.g., sensitivity or subgroup analyses, meta-regression [see Item 16]). | NA. |
| **DISCUSSION** | |  | |  |  |
| Summary of evidence | | 24 | | Summarize the main findings including the strength of evidence for each main outcome; consider their relevance to key groups (e.g., healthcare providers, users, and policy makers). | 6-8. |
| Limitations | | 25 | | Discuss limitations at study and outcome level (e.g., risk of bias), and at review-level (e.g., incomplete retrieval of identified research, reporting bias). | 6-8. |
| Conclusions | | 26 | | Provide a general interpretation of the results in the context of other evidence, and implications for future research. | 6-8. |
| **FUNDING** | |  | |  |  |
| Funding | | 27 | | Describe sources of funding for the systematic review and other support (e.g., supply of data); role of funders for the systematic review. | 9. |
